# Effect of Shot Peening Pretreatment on the Fatigue Behavior of AA5052/SPFC440 Self-Piercing Riveted Joints

**DOI:** 10.3390/ma19061084

**Published:** 2026-03-11

**Authors:** Zejie Zhou, Xiang Ji, Zhichao Huang, Xushuai Gu, Yongchao Zhang

**Affiliations:** School of Materials Science and Engineering, East China Jiaotong University, Nanchang 330013, China; cancyzhou@163.com (Z.Z.); qq1055478690@163.com (X.J.); 17763468692@163.com (X.G.); bzzychao@163.com (Y.Z.)

**Keywords:** self-piercing riveting, shot peening, aluminum–steel joint, fatigue behavior, residual compressive stress

## Abstract

Fatigue properties remain a key challenge for aluminum–steel self-piercing riveted (SPR) joints in lightweight structures. This study evaluates shot peening as a pretreatment for the AA5052 sheet to improve the fatigue behavior of AA5052/SPFC440 dissimilar joints and to clarify the underlying mechanisms. Shot-peened and conventional SPR joints were prepared for comparison. Quasi-static tensile tests were conducted, and tension–tension fatigue tests were performed at high and low load levels. After shot peening, multiple factors with residual compressive stress, subsurface hardening, and surface roughness influenced the fatigue performance of the SPR joints. This led to a load-level-dependent fatigue behavior, with improved fatigue performance at low load levels and reduced performance at high load levels. At high load conditions, the increased surface roughness played a more significant role, with more crack initiation sites observed, resulting in fatigue lives comparable to or slightly lower than those of conventional joints. In contrast, at low load levels in the long-life regime, surface tensile stress was effectively reduced, crack initiation at surface defects was suppressed, and crack initiation shifted from the surface to subsurface regions, resulting in an 11.3% improvement in fatigue strength. These findings provide practical guidance for improving the fatigue performance of dissimilar-material SPR joints through material surface pretreatment.

## 1. Introduction

Vehicle lightweighting has become a major trend in the automotive industry. The adoption of lightweight structural materials, such as aluminum alloys and advanced high-strength steels, is therefore a key approach to reducing body mass and improving fuel efficiency and driving range [1]. However, reliable joining of dissimilar materials remains a technical challenge. Conventional welding processes tend to cause thermal distortion and generate brittle phases, making it difficult to achieve high joint strength [2].

Self-piercing riveting (SPR) is a cold-forming joining process. During SPR, a rivet pierces the upper sheet and flares within the lower sheet to form a mechanical interlock, enabling robust aluminum–steel joining without pre-drilling [3,4]. Enhancing the properties of SPR joints remains a key research focus. Improving joint quality is typically prioritized by optimizing SPR process parameters, such as die design, sheet thickness, and rivet dimensions [5,6]. By combining experiments and numerical simulations, Zhao et al. [7] found that the top-sheet thickness was strongly positively correlated with joint quality, whereas the bottom-sheet thickness showed a pronounced negative correlation; in contrast, the effect of rivet length on joint quality was relatively limited. For dissimilar-material joints, the sheet stacking sequence is a key factor in the riveting process. Wang et al. [8] examined how stacking configuration (steel–aluminum, SA; aluminum–aluminum, AA; aluminum–steel, AS) interacts with rivet hardness (H4 vs. H6) in SPR joints and reported that H4 was suitable for AA, whereas H6 was preferable for SA and AS.

For riveting a metal sheet to a composite laminate, the metal sheet is typically placed as the top layer, whereas the composite laminate is positioned as the bottom layer [9]. Although pre-drilling is not necessary for riveting, existing studies indicate that it can effectively improve riveting properties. Wang et al. [10] investigated the effects of pre-drilled hole diameter and sheet thickness on the quasi-static and fatigue properties of high-strength aluminum–steel SPR joints. Their results showed that fatigue life decreased with increasing hole diameter but increased with increasing sheet thickness. Pre-drilling facilitates the flow of adhesive into the rivet cavity, thereby creating an additional bond between the rivet and the upper and lower sheets [11,12].

In addition to optimizing SPR process parameters to improve joint quality, an increasing number of researchers have recently explored hybrid joining approaches that combine SPR with other processes, such as electromagnetic self-piercing riveting (E-SPR) [13,14,15,16], friction self-piercing riveting (F-SPR) [17,18,19,20,21], and thermal self-piercing riveting (T-SPR) [22,23]. Electromagnetic self-piercing riveting (E-SPR) combines electromagnetic forming with SPR. A strong pulsed magnetic field generated by rapid discharge accelerates the rivet under electromagnetic force, enabling it to pierce the sheets and form a mechanical interlock. Wen et al. [13] investigated the mechanical response of CFRP–aluminum joints produced by E-SPR under different loading rates. The results showed that the CFRP mainly exhibited compressive strain at low loading rates, whereas increasing the loading rate led to more localized strain that propagated rapidly, ultimately causing fracture in the tensile loading direction. In joints involving composite materials, adhesive bonding–riveting hybrid joining is often employed because FRP materials exhibit low ductility [24,25,26]. Friction self-piercing riveting (F-SPR) is an advanced joining technique that integrates mechanical fastening with friction welding, enabling the joining of metals to thermoplastic composites. In F-SPR, a high-speed rotating rivet generates frictional heat under axial pressure, leading to localized plasticization and mechanical anchoring [27]. Zhang [21] and Yang [17] employed flat-die-based F-SPR processes and observed dynamic recrystallization as well as fine-grained zones. They identified these microstructural features as key contributors to joint strengthening. In a study on F-SPR joints, Ma et al. [28] reported a clear requirement for rivet pull-out to dominate. This failure mode relied on three conditions: a hard rivet, a larger rivet flare, and solid-state bonding within the rivet shank. Under this combination, both the peak load and energy absorption increased. For some high-strength metals with limited ductility, T-SPR improves formability through localized heating, thereby delaying crack initiation and propagation. Zhuang et al. [29] found that higher riveting temperatures suppressed delamination growth in CFRP laminates and altered damage evolution. This effect was most evident near the glass transition temperature, where the delamination crack length decreased sharply and the joint quality improved.

Shot peening (SP), as a surface-strengthening process, can enhance the surface performance of materials by introducing residual compressive stress and near-surface plastic deformation [30,31]. Previous studies have shown that SP can influence fatigue crack initiation by modifying the local stress–microstructure state [32,33,34]. Its effect on fatigue performance is load-level dependent: residual compressive stress generally delays crack initiation, whereas increased surface roughness may intensify stress concentration [35,36]. Consequently, fatigue life may improve at low load levels but show limited or even adverse effects at high load levels. The fatigue behavior of structural components is strongly influenced by local strain distribution and its evolution under cyclic loading. Strain–life approaches developed for structural members [37] indicate that load-level dependency and strain localization play decisive roles in crack initiation and fatigue life prediction. These considerations further emphasize the necessity of distinguishing fatigue behavior under different loading conditions when evaluating surface modification strategies.

In our previous work [38], it was demonstrated that shot peening could improve the fatigue strength of aluminum–aluminum SPR joints. However, the load-level-dependent fatigue response and the evolution mechanism of crack initiation behavior have not yet been sufficiently clarified. In particular, the respective roles of residual compressive stress, subsurface hardening, and surface roughness under different cyclic load levels have not been systematically examined. Furthermore, for AA5052/SPFC440 SPR joints, fatigue cracks predominantly initiate on the aluminum side [39]. We therefore consider that regulating the near-surface condition of the aluminum sheet through SP pretreatment may provide a targeted means to influence crack initiation in the most fatigue-critical region of this joint system. Accordingly, this study systematically investigates the fatigue behavior of shot-peened AA5052/SPFC440 SPR joints under both high and low load conditions. Special attention is given to load-level-dependent behavior and variations in crack initiation characteristics. By correlating residual stress distribution, microhardness variation, surface roughness, and fatigue performance of SPR joints, this work aims to provide a clearer understanding of how surface pretreatment influences the fatigue behavior of dissimilar-material SPR joints.

## 2. Materials and Methods

### 2.1. Materials

In this study, AA5052 aluminum alloy sheets (2.5 mm) were used as the upper sheet, and SPFC440 high-strength steel (2.0 mm) as the lower sheet. Table 1 presents the mechanical properties of the AA5052 alloy and SPFC440 steel, respectively, as provided by the suppliers.

### 2.2. Methods

Shot peening was performed using a pressure-fed 1212 system (Kunshan Carter Precision Engineering Co., Ltd., Kunshan, China). Double-sided peening was applied using 0.3 mm glass beads, an Almen intensity of 0.2 mmN, and 100% coverage. Given that fatigue failure has been reported to occur predominantly on the AA5052 side for this joint system, only the aluminum sheet was treated [39]. The residual compressive stress depth profile of shot-peened aluminum sheets was characterized using an LXRD X-ray stress measurement system (Proto Manufacturing Ltd., Windsor, ON, Canada). During the measurements, the tube voltage and current were set to 30 kV and 25 mA, respectively. Residual stresses were evaluated on the Al (311) diffraction plane. To obtain the subsurface stress distribution, a layer-by-layer material removal approach based on electropolishing was employed. The removed depth was monitored using a digital micrometer. Residual stresses were calculated using the sin^2^ψ method. Each stress value represents the average of three independent measurements at the same depth position. The experimental uncertainty was within ±15 MPa, as determined from instrument calibration and repeatability assessment. The 3D surface topography and roughness of the shot-peened and non-shot-peened surfaces were characterized using a Rtec Rev. 2.0 system (Rtec Instruments Inc., San Jose, CA, USA) equipped with a white-light interferometry module. Surface and cross-sectional microhardness measurements were carried out using a Qness 10A+ Vickers microhardness tester (Qness GmbH, Golling an der Salzach, Austria). A load of 10 g was applied for all indentations, with a dwell time of 10 s. The specimens were sectioned, mounted and polished prior to microhardness testing. Hardness values were recorded as a function of the distance from the treated surface.

**Figure 1:**
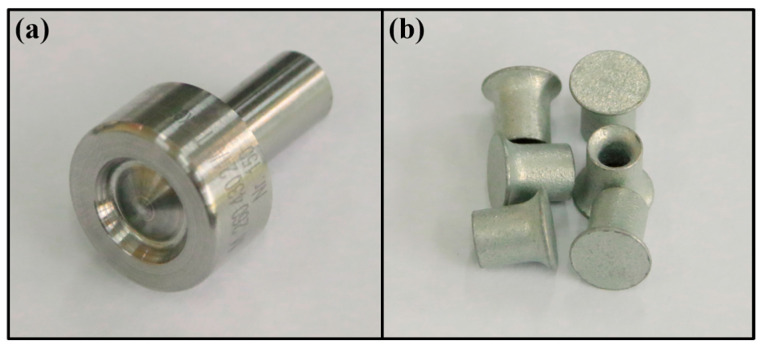
The die and rivet used in this work: (**a**) Boss-concave die; (**b**) Semi-tubular rivet.

The boss-concave die and semi-tubular rivet used in this study are shown in Figure 1. The rivets have a diameter of 5 mm and a length (height) of 7 mm.

**Figure 2:**
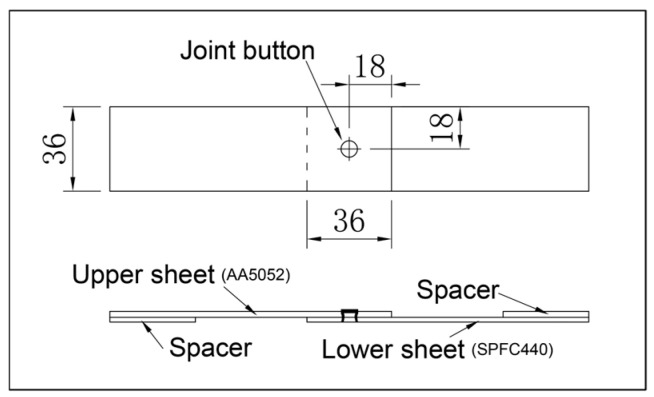
The size of the lap-shear SPR joint [39].

**Figure 3:**
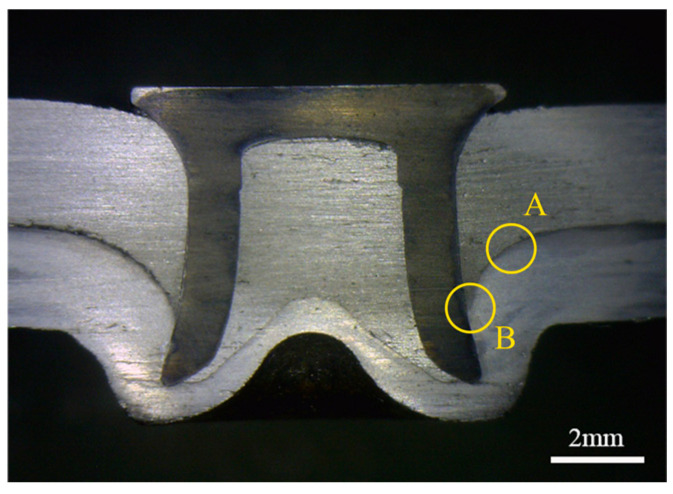
Cross-section of the SPR joint. Position A denotes the contact interface between the steel and aluminum sheets, while Position B indicates the tri-material contact region among the steel sheet, aluminum sheet, and rivet.

SPR was performed using a battery-powered handheld unit (RV300023, Henrob Ltd., Flintshire, UK). The dimensions of the riveted specimens are shown in Figure 2. A cross-sectional micrograph of a representative SPR joint is shown in Figure 3, illustrating the deformation of the semi-tubular rivet and the mechanical interlock between the rivet and the sheets. The joints shown were used for quasi-static tensile and fatigue tests.

Quasi-static tensile tests were performed using a universal testing machine (RGM4030, Shenzhen Reger Instrument Co., Ltd., Shenzhen, China) at a crosshead speed of 2 mm/min. To minimize the bending moment during gripping, spacers with matching thickness were placed in the grip sections on both sides, as shown in Figure 2. Fatigue tests on the SPR joints were carried out using a computer-controlled high-frequency fatigue testing machine (QBG-50, Changchun Qianbang Testing Equipment Co., Ltd., Changchun, China).

Fatigue tests were conducted under tension–tension loading at a frequency of 85 Hz with a stress ratio of 0.1. The stress ratio was defined as R = *P*_min_/*P*_max_, where *P*_max_ and *P*_min_ are the maximum and minimum cyclic loads, respectively. Fatigue life, *N*_f_, was defined as the number of cycles to failure. If no fracture occurred within 1 × 10^7^ cycles, the specimen was recorded as a run-out, and the test was terminated. Lower load levels were not further examined. To efficiently determine the fatigue life range and the run-out window, the load levels were selected using a stepwise approach rather than fixed load increments. The fracture surface morphology was characterized by SEM (JSM-6360LA, JEOL Ltd., Tokyo, Japan).

## 3. Results and Discussion

This section elucidates how shot peening modifies the surface integrity of AA5052 sheets, in terms of residual compressive stress, surface topography and roughness, and microhardness, and how these surface states control the fatigue behavior of SPR joints.

### 3.1. Effects of Shot Peening on Surface Characteristics of AA5052 Sheets

#### 3.1.1. Residual Compressive Stress Profile

**Figure 4:**
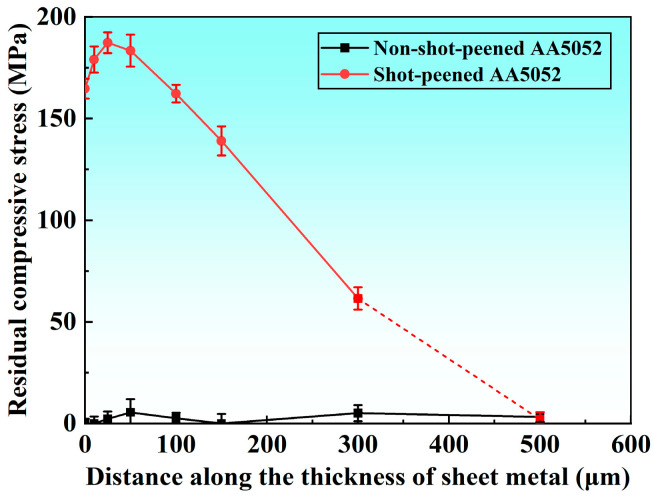
Residual compressive stress distribution in the thickness of the sheets.

Figure 4 presents the depth profile of residual compressive stress in the AA5052 sheet. The non-shot-peened sheet showed negligible compressive stress near the surface, whereas shot peening introduced a pronounced compressive stress layer extending to at least 300 μm in depth. The compressive stress increased with depth to a subsurface maximum and then decreased, peaking at approximately 187 MPa at a depth of 20–40 μm.

The formation of a residual compressive stress layer in the surface region of the aluminum sheet after shot peening was mainly attributed to the impact mechanism and the material’s elastoplastic response. During shot peening, high-velocity shots struck the surface and converted their kinetic energy into plastic deformation work in the surface layer, causing localized compressive plastic strain. Owing to the high ductility of aluminum, shot impacts promoted plastic flow in the surface layer. Local material was compressed and laterally displaced, leaving microscopic indentations. The plastic deformation activated lattice slip, and dislocations accumulated at grain boundaries or second-phase particles, leading to local stress fields. After the impacts ceased, the plastically deformed surface layer was elastically constrained by the underlying material, which underwent little plastic deformation and therefore could not fully recover. As a result, compressive strain was retained in the surface layer, ultimately generating macroscopic residual compressive stress.

The residual compressive stress along the sheet thickness direction first increased and then decreased, with the maximum value occurring in the subsurface layer rather than at the outermost surface. This trend was mainly associated with the degree of surface plastic deformation and the attenuation of impact energy with depth. During shot impacts at high velocity, the impact energy was rapidly dissipated in the surface layer. The surface directly sustained the impacts and underwent severe plastic deformation; however, because it was a free boundary, it could not develop a high level of constraint stress. With increasing depth, the impact of kinetic energy gradually attenuated, while the subsurface region experienced stronger constraints on multidirectional plastic flow, causing the residual compressive stress to peak in the subsurface layer (at a depth of several tens of micrometers). Surface plastic deformation triggered stress redistribution within the material. The subsurface region experienced higher compressive stress due to the extrusion associated with surface plastic flow, whereas the deeper region provided an elastic counter-constraint to the deformation of the upper layer, thereby establishing stress equilibrium. This elastoplastic coupling caused the maximum residual compressive stress to occur in the subsurface region rather than at the immediate surface. It should be noted that XRD-based residual stress measurements in gradient stress fields may be affected by stress relaxation during layer removal. In the present study, incremental material removal with small step sizes was adopted to minimize such effects. The consistency of the subsurface compressive peak across repeated measurements confirms the reliability of the observed stress distribution.

#### 3.1.2. Surface Topography and Roughness

**Figure 5:**
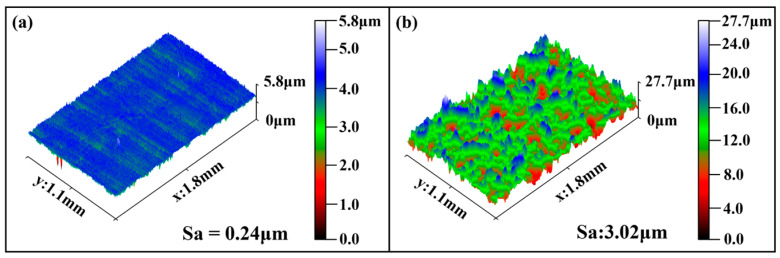
3D surface topography of AA5052 sheets: (**a**) non-shot-peened and (**b**) shot-peened.

The surface of the non-shot-peened AA5052 was relatively smooth, as shown in Figure 5a. Only shallow machining marks and small height variations were observed. The peak-to-valley height was about 5.8 μm, and the corresponding Sa (arithmetical mean height) value was 0.24 μm, indicating low surface roughness and limited geometric discontinuities. The surface morphology changed drastically after shot peening (Figure 5b). Numerous impact craters were formed by high-velocity shots, and pronounced plastic uplift developed around the craters. These features combined to produce a densely distributed peak–valley structure. The peak-to-valley height increased to approximately 27.7 μm and Sa rose sharply to 3.02 μm, indicating a strong increase in surface undulation. This roughened morphology originated from severe near-surface plastic deformation induced by repeated shot impacts. Within the impact zones, the material experienced indentation, lateral flow, and constrained rebound. These deformation processes caused material accumulation and bulging around the craters. Although shot peening was beneficial in introducing high dislocation density and residual compressive stresses in the subsurface layer, it also generated pronounced geometric discontinuities and stress concentration sites at the surface. From a surface-integrity standpoint, shot peening introduced two competing effects: mechanical strengthening through residual compressive stress and work hardening, and geometric weakening due to increased roughness and peak–valley topography. This dual effect directly governed the stress distribution and damage evolution in the surface layer under cyclic loading, and formed the physical basis for the load-dependent fatigue behaviour of the shot-peened joints.

#### 3.1.3. Surface Microhardness

**Figure 6:**
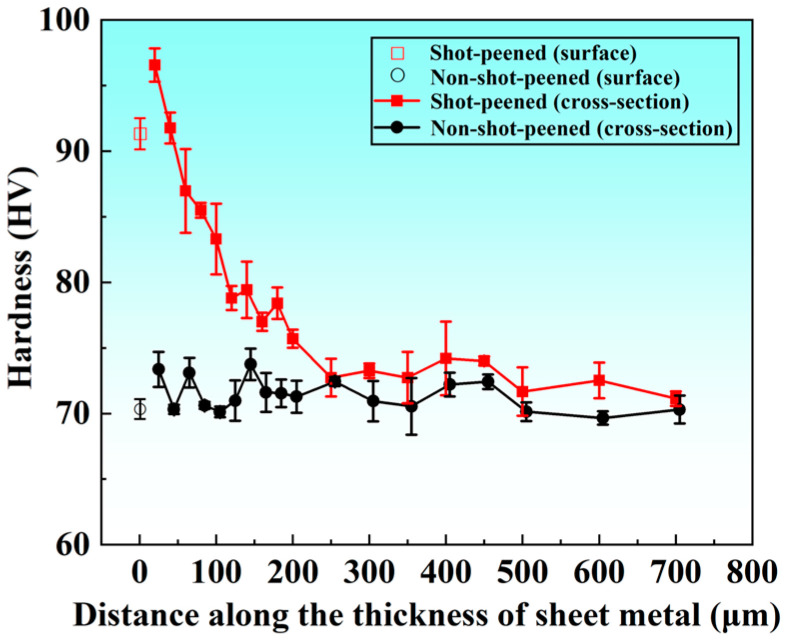
Microhardness distribution in the thickness of the sheets.

The microhardness of the non-shot-peened AA5052 remained relatively stable, as shown in Figure 6, with both the surface and cross-sectional values lying in the range of about 70–73 HV. No obvious surface-hardened layer was observed. In contrast, shot peening markedly altered the hardness distribution. The surface microhardness of AA5052 increased significantly to about 91 HV, which represented an improvement of approximately 30% compared with the untreated material. In addition, a pronounced hardness gradient developed along the depth direction. The maximum hardness did not occur at the outermost surface but reached about 97 HV at a subsurface depth of approximately 20 μm. With increasing depth, the hardness gradually decreased and fell to the bulk level at depths beyond about 250 μm. This indicated that the hardening induced by shot peening was mainly confined to the near-surface region.

Since shot impacts induced non-uniform plastic deformation and the associated work-hardening effect, the near-surface hardness increased after shot peening. The surface layer underwent intense plastic shearing and compressive deformation under high-strain-rate impacts. This promoted dislocation accumulation and increased the resistance to plastic deformation, which was reflected by the higher microhardness. The maximum hardness occurred in the subsurface region (at a depth of about 20 μm) rather than at the outermost surface. This was attributed to the weaker constraint at the free surface and the stronger confinement in the subsurface, which promoted a higher level of plastic deformation and work hardening. In addition, local strain relaxation at the outermost surface could make the surface hardness slightly lower than that in the subsurface. The depth of the hardness peak was consistent with the region where the peak compressive residual stress was observed (20–40 μm). With increasing depth, the impact-induced strain rapidly attenuated and the work-hardening effect weakened. Therefore, the hardness gradually decreased toward the bulk level.

### 3.2. Effect of Shot Peening on Fatigue Behavior of SPR Joints

#### 3.2.1. Quasi-Static Tensile Load of SPR Joints

Tensile tests were performed on AA/SPFC-A joints prepared by conventional SPR and AA/SPFC-B joints prepared by the SP–SPR hybrid process, with three joints tested in each group. The tensile results are shown in Table 2, respectively, where *P*_b_ denotes the quasi-static tensile failure load.

As shown in Table 2, the quasi-static tensile failure load decreased from 7.76 kN (AA/SPFC-A) to 7.35 kN (AA/SPFC-B) after shot peening, corresponding to a reduction of approximately 5.3%. The average values were obtained from three specimens per condition, with standard deviations of 0.17 kN and 0.09 kN, respectively. The magnitude of the reduction is larger than the typical experimental scatter, suggesting that the observed decrease is systematic rather than purely random. This decrease is consistent with the roughening effect of shot peening, which introduces surface indentations and increases local stress concentration, thereby promoting earlier damage under monotonic loading.

#### 3.2.2. Fatigue Testing Results and Discussion

The fatigue lives obtained under different maximum loads are summarized in Table 3. Overall, both joint types showed a marked increase in fatigue life as the maximum load decreased. Under a maximum load of 6.0 kN, the fatigue lives of AA/SPFC-A and AA/SPFC-B were 51.4 × 10^3^ and 41.8 × 10^3^ cycles, respectively. Reducing the maximum load to 5.2 kN and 4.5 kN increased the lives of both groups to the order of 10^5^ cycles. With a further reduction to 3.0 kN, the lives reached 1964.4 × 10^3^ cycles for AA/SPFC-A and 4752.3 × 10^3^ cycles for AA/SPFC-B. At lower load levels, run-out was observed. Specifically, AA/SPFC-B survived 1 × 10^7^ cycles without failure at 2.9 kN and 2.8 kN. For AA/SPFC-A, run-out was achieved at 2.6 kN, whereas fatigue lives of 4027.4 × 10^3^ and 7513.3 × 10^3^ cycles were obtained at 2.8 kN and 2.7 kN, respectively.

Based on the fatigue data summarized in Table 3, the *P*_max_–*N*_f_ scatter plots were constructed for both joint types. The data were fitted using a bilogarithmic regression of the standard form log_10_ (*N*_f_) = a + b log_10_ (*P*_max_). Only failure data were included in the regression analysis, whereas run-out data (no failure up to 10^7^ cycles) were excluded. The coefficients of determination (R^2^) for AA/SPFC-A and AA/SPFC-B were 0.983 and 0.976, respectively, indicating a strong linear correlation in bilogarithmic coordinates. Owing to the limited number of specimens, the fitted curves are intended to describe the overall fatigue trend rather than to establish statistically definitive fatigue limits. Nevertheless, the additional tests at intermediate load levels show consistent fatigue-life differences between the two variants without large outliers, suggesting that the observed improvement is systematic rather than random. The resulting fitted relationships for AA/SPFC-A and AA/SPFC-B are given in Equations (1) and (2), respectively.

log_10_ (*N*_f_) = 9.33 − 6.03 log_10_ (*P*_max_) (1)

log_10_ (*N*_f_) = 10.20 − 7.18 log_10_ (*P*_max_) (2)

The 5% criterion is an interval-based method that estimates fatigue strength from the adjacent run-out and failure load levels [40]. It is complementary to the regression-based S–N description and does not rely on extrapolating the fitted S–N line to define a fatigue limit. According to the 5% criterion, the fatigue strength is determined as the midpoint between the highest load level producing run-out (*P*_ro_) and the lowest load level leading to failure (*P*_f_):

(3) $$P_{f,0.05}=\frac{P_{f}+P_{ro}}{2}$$

For both joint types, the adjacent failure and run-out load levels summarized in Table 3 were substituted into Equation (3) to estimate the fatigue strength. The resulting values are given in Equations (4) and (5).

The uncertainty associated with the 5% criterion estimation can be bounded within ±ΔP/2. In the present case, ΔP = 0.1 kN, leading to an interval-related uncertainty of approximately ±0.05 kN. This uncertainty is smaller than the observed difference in fatigue strength between the two variants (0.30 kN), indicating that the improvement induced by shot peening pretreatment exceeds the interval-induced estimation uncertainty.

(4) $$P_{f,0.05}=\frac{2.7+2.6}{2}=2.65\;kN$$

(5) $$P_{f,0.05}=\frac{3.0+2.9}{2}=2.95\;kN$$

Based on the above estimation, shot peening pretreatment increased the fatigue strength from 2.65 kN to 2.95 kN (approximately 11.3%). Given the limited number of specimens, this enhancement should be interpreted as an indicative improvement under the tested conditions.

**Figure 7:**
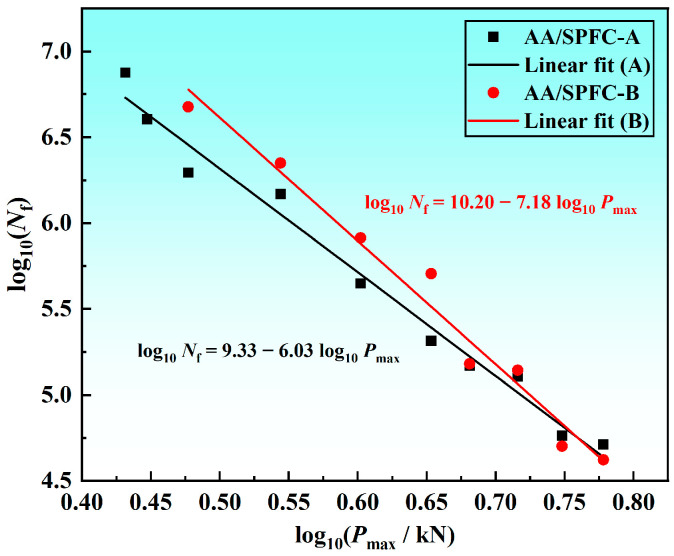
*P*_max_–*N*_f_ curves in bilogarithmic coordinates. Note: The regression was performed using failure data only (run-out at $10^{7}$ cycles excluded); the number of failure points used for fitting is indicated in Table 3.

As shown in Figure 7, the fatigue lives of the AA/SPFC-A joints were comparable to, or in some cases longer than, those of the AA/SPFC-B joints at high load levels (*P*_max_ ≥ 5.2 kN).

By contrast, in the long-life regime at lower loads (represented by *P*_max_ ≤ 4.5 kN), the AA/SPFC-B joints exhibited longer fatigue lives than the AA/SPFC-A joints. These results suggested that shot peening provided a more pronounced benefit in the long-life regime.

#### 3.2.3. Analysis of Fatigue Failure Mode

**Figure 8:**
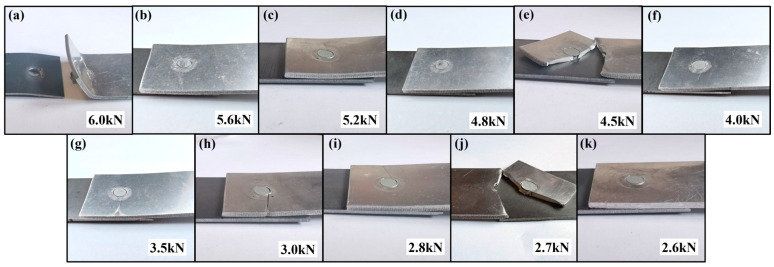
Deformation characteristics of AA/SPFC-A joints under different maximum loads: (**a**) 6.0 kN; (**b**) 5.6 kN; (**c**) 5.2 kN; (**d**) 4.8 kN; (**e**) 4.5 kN; (**f**) 4.0 kN; (**g**) 3.5 kN; (**h**) 3.0 kN; (**i**) 2.8 kN; (**j**) 2.7 kN; (**k**) 2.6 kN.

**Figure 9:**
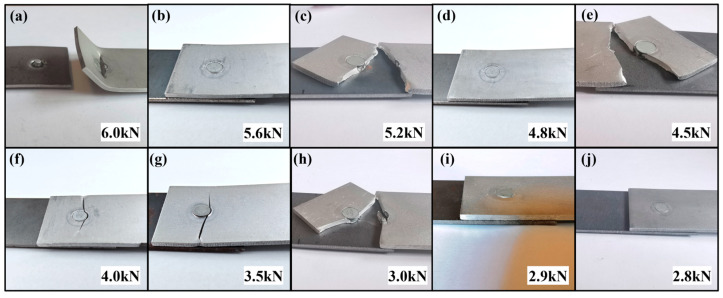
Deformation characteristics of AA/SPFC-B joints under different maximum loads: (**a**) 6.0 kN; (**b**) 5.6 kN; (**c**) 5.2 kN; (**d**) 4.8 kN; (**e**) 4.5 kN; (**f**) 4.0 kN; (**g**) 3.5 kN; (**h**) 3.0 kN; (**i**) 2.9 kN; (**j**) 2.8 kN.

Fatigue failure modes of the AA/SPFC-A joints and the AA/SPFC-B joints at different load levels are shown in Figure 8 and Figure 9, respectively. Cracks occurred on the aluminum sheet side for all specimens that experienced fatigue failure. The crack was located at the rivet edge on the fixture side of the aluminum sheet, and it initiated in the vicinity of the rivet head before propagating toward both sides of the sheet.

#### 3.2.4. Analysis of Fatigue Failure Mechanisms

At higher load levels, the upper sheet exhibited pronounced out-of-plane warping, as shown in Figure 8a and Figure 9a. The aluminum sheet exhibited severe out-of-plane warping at 6.0 kN. The rivet tail pulled out from the lower sheet, resulting in separation between the upper and lower sheets, while the rivet remained embedded in the upper sheet. At lower load levels, only slight or even negligible out-of-plane warping was observed. This behavior was mainly associated with the mean load in the fatigue cycle (i.e., the average of the maximum and minimum loads). Under the present loading condition (R = 0.1), the mean load increased with the maximum load. When the load was 6 kN, the mean load was approximately 3.3 kN. The higher mean load promoted tensile-dominated deformation and geometric instability of the aluminum sheet, leading to pronounced out-of-plane warping under high-load fatigue. Although the macroscopic deformation morphology became more severe at high loads, the fatigue failure mechanism remained governed by cyclic damage accumulation rather than monotonic fracture.

Fatigue cracks nucleated in the aluminum sheet due to various initiating factors and propagated under cyclic loading until final failure. Meanwhile, the relatively high mean load component also caused a certain degree of plastic deformation. At maximum loads below 3.0 kN, the mean load remained below 1.65 kN and no noticeable tensile-like out-of-plane warping was observed; failure was therefore dominated by fatigue crack initiation and growth under cyclic loading.

Because the effect of shot peening on SPR joints was load-level dependent, the failure mechanisms in the high-load and low-load regimes were analyzed separately.

1. Fatigue failure mechanisms under high-stress amplitude loading

**Figure 10:**
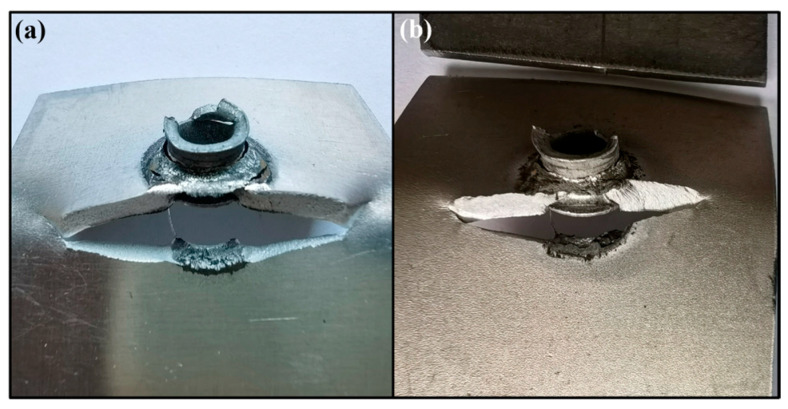
Fatigue cracks on the aluminum sheets of SPR joints: (**a**) AA/SPFC-A; (**b**) AA/SPFC-B.

To elucidate the fatigue failure mechanism of shot-peened joints, the Al/steel contact interface was examined, and AA/SPFC-A and AA/SPFC-B joints were compared under a fatigue load of 6.0 kN. Figure 10 shows the fracture surface morphology of the aluminum sheets, where Figure 10a and Figure 10b correspond to the fatigue cracks of the AA/SPFC-A and AA/SPFC-B joints, respectively.

Under tension–tension fatigue at high load levels, the joints were subjected to high tensile stresses and exhibited a certain degree of plastic deformation under a relatively high mean load. The combination of the applied tensile stress and the local stress concentration is believed to play a significant role in contributing to the fatigue failure of the riveted joints at high load levels. Additionally, interfacial wear may have further facilitated crack nucleation. Since fatigue testing was conducted immediately after riveting, the contact duration between aluminum and steel was short, and the effect of electrochemical corrosion was expected to be limited.

**Figure 11:**
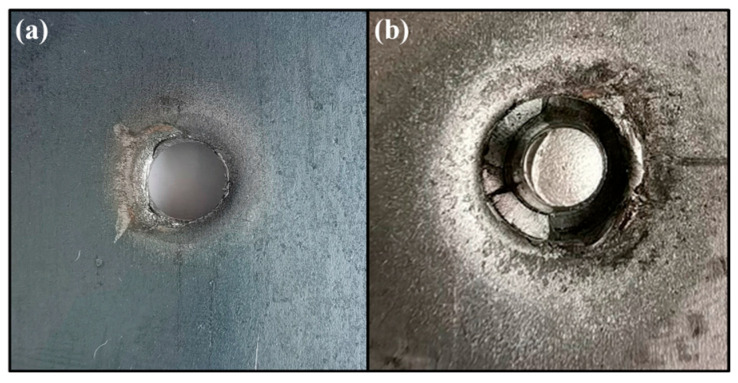
Fatigue cracks on the steel sheets of SPR joints: (**a**) AA/SPFC-A; (**b**) AA/SPFC-B.

Figure 11 shows the fatigue fracture features on the steel sheet. For the AA/SPFC-A joints, the button-like protrusion fractured completely and pulled out from the steel sheet. In contrast, the protrusion in the AA/SPFC-B joints remained intact, and fragments from the fractured rivet tail were retained in the button recess.

As shown in Table 3 and Figure 7, at high load levels, the fatigue lives of the AA/SPFC-A joints were comparable to, or even longer than, those of the AA/SPFC-B joints. The corresponding failure modes indicate that the relatively high mean load in the fatigue cycle led to pronounced out-of-plane deformation of the sheets, which modified the local stress state around the rivet. The combined effects of applied tensile stress, bending-induced tensile residual stress, and local stress concentration became dominant in governing crack initiation and propagation. In this regime, the contribution of SP-induced residual compressive stress was relatively reduced. Meanwhile, surface roughness and dimples introduced by shot peening further intensified local tensile stress by reducing the effective load-bearing area. As a result, shot peening did not provide a clear fatigue-strength benefit at high load levels.

2. Fatigue failure mechanisms under low-stress amplitude loading

**Figure 12:**
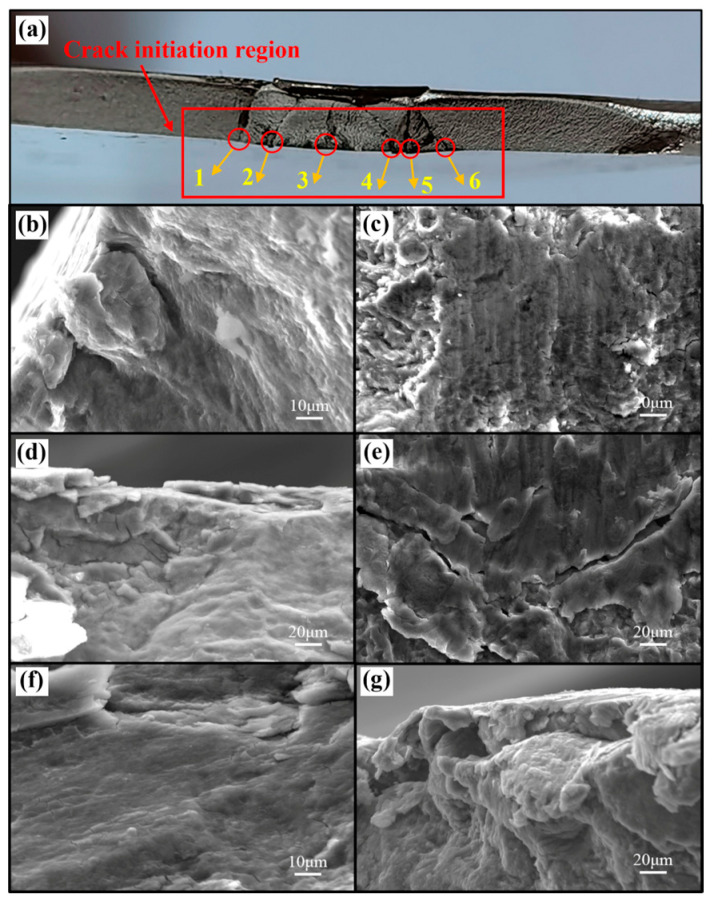
Aluminum-side fatigue fracture morphology of the AA/SPFC-A joint at 3 kN: (**a**) macroscopic view of the aluminum-sheet fracture surface showing the fatigue crack and the crack-initiation sites (1–6); (**b**–**g**) corresponding SEM images taken from sites 1–6 in (**a**).

**Figure 13:**
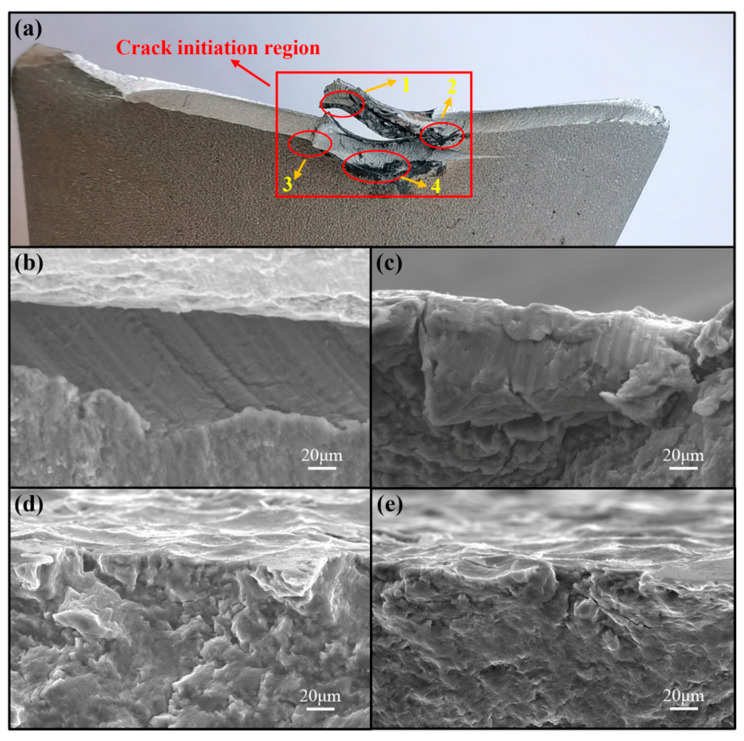
Aluminum-side fatigue fracture morphology of the AA/SPFC-B joint at 3 kN: (**a**) macroscopic view of the aluminum-sheet fracture surface showing the fatigue crack and the crack-initiation sites (1–4); (**b**–**e**) corresponding SEM images taken from sites 1–4 in (**a**).

Taking the joints tested at 3.0 kN as a representative case, the fatigue failure mechanism of the SPR joints was analyzed. The aluminum-side fatigue fracture morphologies of the AA/SPFC-A and AA/SPFC-B joints are shown in Figure 12 and Figure 13, respectively.

As shown in Figure 12a and Figure 13a, the aluminum-side fatigue fracture morphologies of both AA/SPFC-A and AA/SPFC-B joints exhibit multiple crack-initiation sites. For the AA/SPFC-A joints, at least six crack-initiation sites can be identified, with the corresponding local features shown in Figure 12b–g. In contrast, fewer initiation sites (four in total) are observed for the AA/SPFC-B joints, as shown in Figure 13b–e. This difference suggests that SP pretreatment may reduce the tendency for crack initiation and modify the crack nucleation behavior.

As shown in Figure 13d,e, fatigue cracks in the shot-peened joints predominantly originated from subsurface regions, whereas cracks in the AA/SPFC-A joints initiated at the free surface (Figure 12b,d,g). This transition in dominant crack initiation location reflects a modification of the local stress–microstructure interaction rather than a simple strengthening effect. Shot peening introduces a strain-hardened surface layer and residual compressive stress, both of which reduce the effective tensile stress at the free surface. However, the riveting process may lead to local stress redistribution and potentially introduce tensile residual stress in subsurface regions due to bending deformation. As a result, when surface crack initiation is restrained, the subsurface region—particularly near the rivet-affected zone—becomes the preferential crack nucleation site.

Fatigue crack initiation sites in the non-shot-peened aluminum sheet are located at Position A in Figure 3. Cracks initiated mainly from Sites 1 and 6, propagated toward both ends of the sheet, and eventually led to the final fracture. For the AA/SPFC-B joints, crack initiation Sites 3 and 4 are located at Position A in Figure 3, whereas Sites 1 and 2 are located at Position B. During crack growth, fracture morphology features suggest coalescence between the regions labeled Sites 1 and 3, followed by preferential propagation toward one side of the sheet. On the opposite side, propagation features associated with Site 2 became more pronounced and extended toward the sheet edge. Under cyclic fatigue loading, pronounced interfacial wear was observed at the fracture region, and fatigue cracks in the aluminum sheet initiated from these wear-affected locations. During cyclic loading, slight relative motion at the Al/steel interface likely resulted in local interfacial wear. The repeated micro-sliding may mechanically expose fresh metal surfaces, where thin oxide films can form in the presence of oxygen and moisture. The rupture of such films and the generation of wear debris may further aggravate local surface damage. The accumulated debris subsequently pulled out and produced wear pits. Meanwhile, the wear-affected region on the upper sheet was subjected to relatively high tensile stress under the applied load. Because the wear pits coincided with this high-stress region, fatigue cracks nucleated within the pits. In addition, the sheet at the wear location underwent downward bending deformation due to the rivet penetration force during riveting, which may alter the local stress state in the subsurface layer. The wear-affected regions likely acted as stress concentrators under cyclic loading. Fatigue crack initiation was thus facilitated by the combined effects of interfacial wear, residual tensile stress, and the applied tensile stress. Since severe electrochemical corrosion in air generally requires a relatively long exposure time, its contribution was expected to be secondary.

At lower load levels, shot peening modified the primary crack nucleation sites. For the AA/SPFC-B joints, cracks predominantly initiated at Positions A and B in Figure 3, whereas for the AA/SPFC-A joints, primary cracks primarily initiated at Position A. During riveting, the aluminum sheet experienced bending-induced tensile deformation at Position A, which altered the local stress state. Under prolonged cyclic loading, this region was repeatedly subjected to interfacial contact and local wear damage, increasing local stress concentration and enhancing the likelihood of crack initiation. This mechanism, observed particularly in the AA/SPFC-A SPR joints, suggests that both shot peening and riveting-induced effects play critical roles in crack nucleation. Qiao et al. [41] reported that shot peening could further refine the surface grains of aluminum alloys and produce a denser subsurface microstructure, which hindered the penetration of corrosive media. As a result, the corrosion current density and corrosion rate were reduced, thereby improving corrosion resistance. This could delay fatigue crack initiation. Moreover, and most importantly, shot peening introduced a residual compressive stress layer on the surface of the aluminum sheet, while a residual tensile stress was retained in the rivet-affected subsurface region due to bending during riveting. This residual compressive stress reduced the effective tensile stress at the surface and lowered the driving force for crack initiation and early propagation, thereby delaying fatigue crack formation. When a fatigue crack initiated at the surface of the aluminum sheet at Position A and tended to propagate into the interior, it was influenced by the surface residual compressive stress, which counteracted part of the applied tensile stress and reduced the driving force for crack growth, thereby hindering crack propagation. Crack-initiation Site 4 in Figure 13a is a representative example of this behavior. At Position B, the aluminum sheet was locally sheared or cut by the rivet during riveting, which relieved the residual compressive stress introduced by shot peening. Meanwhile, the riveting process also led to local thinning and microcracks in the aluminum sheet due to the squeezing effect of the rivet and steel sheet. This region experienced repeated interfacial contact and local wear damage under cyclic loading. Therefore, when crack initiation and growth at Position A were delayed, fatigue cracks initiated at Position B and became dominant crack sources, ultimately leading to fracture, as exemplified by crack-initiation Sites 1 and 2 in Figure 13a. Consequently, at lower load levels, shot peening improved the fatigue properties of the SPR joints by shifting crack initiation to subsurface regions.

## 4. Conclusions

By comparing the conventional SPR joint with the shot-peened SPR joint, this study clarified the effects of shot peening on the fatigue behaviors of AA5052/SPFC440 joints and elucidated the underlying mechanisms. The main conclusions are summarized as follows:

1. A pronounced near-surface residual compressive stress field was introduced by shot peening in the AA5052 sheet. The effective strengthening zone was primarily concentrated within the near-surface region of approximately 200–300 μm, accompanied by an increase in near-surface microhardness of approximately 30%. Notably, the hardness peak occurred in a similar subsurface region to the maximum residual compressive stress. Meanwhile, shot peening increased the Sa value by approximately one order of magnitude, indicating substantial surface roughening.
2. The influence of shot peening on fatigue behavior was load-level dependent. At high load levels, the relatively high mean load induced out-of-plane warping of the sheets and promoted interfacial damage. Meanwhile, shot peening increased the surface roughness, and the associated stress concentration weakened or even offset the beneficial effect of residual compressive stress. Consequently, no improvement in fatigue performance was observed in this regime. The fatigue life of the shot-peened joints was comparable to, or slightly lower than, that of the conventional SPR joints.
3. Shot peening significantly improved the fatigue performance of AA5052/SPFC440 SPR joints under low load conditions, with an increase of approximately 11.3%. Surface modifications induced by shot peening, such as residual compressive stress and subsurface hardening, suppressed the initiation and early propagation of surface cracks, thereby reducing the number of crack initiation sites. Notably, after shot peening, crack initiation sites shifted from the surface to subsurface regions.

## Figures and Tables

**Table 1:** Mechanical properties of AA5052 and SPFC440 [39].

Material	Tensile Strength/MPa	Conditional Yield Strength σ0.2/MPa	Elongation/%
AA5052	200	90	14
SPFC440	440	305	33

**Table 2:** Ultimate tensile load, *P*_b_ (kN).

SPR Technology	Joint 1	Joint 2	Joint 3	Mean ± SD
AA/SPFC-A	7.61	7.95	7.72	7.76 ± 0.17
AA/SPFC-B	7.44	7.36	7.26	7.35 ± 0.09

**Table 3:** Fatigue test data.

Maximum Cyclic Load *P*_max_/kN	Fatigue Life/× 10^3^ cycles	
	AA/SPFC-A	AA/SPFC-B
6.0	51.4	41.8
5.6	57.9	50.3
5.2	128.2	139.2
4.8	147.7	151.4
4.5	206.5	507.9
4.0	445.8	819.3
3.5	1476.5	2237.1
3.0	1964.4	4752.3
2.9	—	≥10,000 (run-out)
2.8	4027.4	≥10,000 (run-out)
2.7	7513.3	—
2.6	≥10,000 (run-out)	—

Note: “≥10,000” indicates run-out (no failure up to 1 × 10^7^ cycles). “—“ indicates that the test was not conducted at this load level.

## Data Availability

The original contributions presented in this study are included in the article. Further inquiries can be directed to the corresponding author.
